# A Multisectoral Emergency Response Approach to a Cholera Outbreak in Zambia: October 2017–February 2018

**DOI:** 10.1093/infdis/jiy490

**Published:** 2018-09-11

**Authors:** Nathan Kapata, Nyambe Sinyange, Mazyanga Lucy Mazaba, Kunda Musonda, Raymond Hamoonga, Muzala Kapina, Khozya Zyambo, Warren Malambo, Ellen Yard, Margaret Riggs, Rupa Narra, Jennifer Murphy, Joan Brunkard, Andrew S Azman, Namani Monze, Kennedy Malama, Jabbin Mulwanda, Victor M Mukonka

**Affiliations:** 1 Ministry of Health, Lusaka, Zambia; 2 Zambia National Public Health Institute, Lusaka; 3 Zambia Field Epidemiology Training Program, Lusaka; 4 US Centers for Disease Control and Prevention, Lusaka, Zambia; 5 Médecins Sans Frontières, Geneva, Switzerland; 6 Johns Hopkins Bloomberg School of Public Health, Baltimore, Maryland; 7 Copperbelt University, School of Medicine, Ndola, Zambia

**Keywords:** multisectoral, response, emergency, cholera, Zambia

Zambia has experienced recurrent cholera outbreaks since the late 1970s, primarily during the rainy season. The 2017–2018 cholera outbreak started on October 6, 2017, initially localized to 2 peri-urban areas of Lusaka, the capital, and was linked to contaminated water consumption. From mid-December 2017 to early February 2018 cases spread city-wide, with 3938 cases and 82 deaths (case fatality rate, 2.1%) by February 17, 2018 (Figure 1).

**Figure 1.**
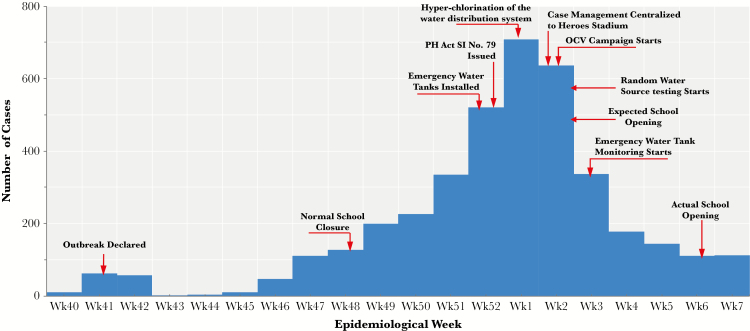
Epidemic curve by week of showing cases of Cholera in Lusaka District, October 2017 to February 2018. PH Act SI No. 79—Public Health Act Statutory Instrument No. 79 of 2017 entitled “The Public Health (Infected Areas) (Cholera) Regulations, 2017”, which led to the following: closure of markets, delayed opening of schools, prohibiting mass gatherings and street vending, and enhanced inspections of premises. Abbreviations: OCV, Oral Cholera Vaccination; Wk, week.

Previous studies in Lusaka documented high levels of contamination of groundwater sources [1, 2] and implicated certain foods in cholera transmission [3, 4]. Inadequate drainage networks have also been associated with cholera incidence in Lusaka [5]. Initial investigations in this outbreak found widespread fecal contamination of water sources (piped water, private boreholes, and shallow wells), household stored water, and market food. Municipal piped water, 60% of which comes from groundwater sources, is intermittently available across Lusaka.

## Emergency Response Activation, Coordination, and Communication

The Ministry of Health (MoH), through the Zambia National Public Health Institute, implemented a multisectoral approach to fighting this epidemic including the Health, Local Government, Education, Water Development, Sanitation, and Environmental Protection sectors with additional support through the National Disaster Management and Mitigation Unit and the national defense wings to support emergency public health interventions.

## Interventions

### Communication

Communication and temporary policy changes played an important role in the cholera response. The dramatic rise in cases occurred during the school holiday when many students temporarily visit Lusaka. To try to limit the geographic expansion of the outbreak beyond Lusaka through travel, the reopening of schools was delayed. A number of markets were closed throughout the city, and street vending of food was prohibited. These closures along with a mass media campaign were meant to increase cholera prevention awareness, to encourage rapid healthcare seeking and to reduce transmission.

### Water Monitoring, Chlorination, and Tank Provision

To ensure adequate supply and access to safe water, 280 new water tanks and 69 extra tap stands were strategically placed in affected areas, serving free disinfected water to approximately 1.2 million people. To improve water safety at the household level, more than 1 million bottles of chlorine and health promotional materials were distributed door to door in high-risk areas. In addition, more than 1500 shallow wells, deemed unsafe, were buried.

A water source-monitoring program to test for residual chlorine levels and *Escherichia coli* in randomly selected households’ water sources was implemented. Of 220 water sources tested during January 2018, 73% had inadequate residual free chlorine (<0.2 mg/L) and 31% were *E coli* positive; boreholes (34%) and shallow wells (91%) were the most contaminated. Daily reports were provided to the water utility company for immediate corrective action.

### Case Management

With the rise in cases, MoH mobilized healthcare professionals to reinforce case management in cholera treatment centers (CTCs) aiming to reduce the case fatality rate to <1%. The CTC assessments and healthcare worker knowledge surveys guided focus areas for mentorship and training. The establishment of a centralized CTC at Heroes National Stadium consolidated case management resources and expertise, which was especially useful for complicated cases.

### Oral Cholera Vaccination Campaign

The Oral Cholera Vaccination (OCV) campaign has been effective in the control of cholera outbreaks in various settings [6]. The MoH targeted 1.2 million people in high-incidence neighborhoods of Lusaka with OCV (Euvichol-Plus) from the global OCV stockpile. The new plastic presentation, in addition to the large number of personnel with experience from the previous year’s OCV campaign and other logistic resources, allowed for a large number of vaccines to be easily transported and stored; in contrast to experience with OCV from a previous outbreak [7], this new presentation allowed for all 1.2 million doses to be delivered within 10 days in the first round. In addition, this new plastic presentation can be taken out of the cold chain on day of administration, which simplifies campaign logistics.

The outbreak presented a number of challenges including inadequate laboratory capacity for culture confirmation, the rapid spread of the outbreak across Lusaka, and the need to quickly train and equip a cholera response workforce. Stigma associated with cholera was also thought to contribute to a delay in seeking healthcare and a high percentage of community deaths.

In response to a rapidly accelerating outbreak, where cases surged from several hundred to several thousand in less than 1 month, the government of Zambia mounted a rapid and robust public health response that included provision of emergency water supplies, water source monitoring and chlorination, enhanced surveillance and epidemiologic investigations, case management training, mass vaccination with significant uptake, community sensitization, and activated a well coordinated emergency response.

## CONCLUSIONS

Cholera outbreaks do not have to occur in Zambia. To achieve a cholera-free Zambia, both short-term and long-term solutions are needed. Zambia recently developed a national multisectoral cholera elimination plan, which encompasses provision of clean water supply, sanitation, community engagement, and health education, in addition to vaccination of more than 2 million people in key cholera hotspots, which is expected to reduce cholera risk immediately; in addition, there are plans for revaccination of hotspots every 3 years. Ultimately, cholera prevention efforts should focus on improving access to safe water through increasing the capacity of the water utility company to meet the needs of all populations, improving sanitation facilities and solid waste management by the Municipal Council, and promoting hygiene behavior change. Finally, enhanced surveillance through Zambia’s Integrated Disease Surveillance and Response will allow outbreaks to be detected early and rapidly contained.

## Notes

***Supplement sponsorship.*** This work is part of a supplement sponsored by the Bill and Melinda Gates Foundation.

***Potential conflicts of interest.*** All authors: No reported conflicts of interest. All authors have submitted the ICMJE Form for Disclosure of Potential Conflicts of Interest. Conflicts that the editors consider relevant to the content of the manuscript have been disclosed.
